# A Voltammetric Biosensor Based on Glassy Carbon Electrodes Modified with Single-Walled Carbon Nanotubes/Hemoglobin for Detection of Acrylamide in Water Extracts from Potato Crisps

**DOI:** 10.3390/s8095832

**Published:** 2008-09-23

**Authors:** Agnieszka Krajewska, Jerzy Radecki, Hanna Radecka

**Affiliations:** Institute of Animal Reproduction and Food Research, The Polish Academy of Sciences, Tuwima 10, 10-747 Olsztyn, Poland

**Keywords:** Acrylamide, hemoglobin, SWCNTs, OSWV, potato crisps

## Abstract

The presence of toxic acrylamide in a wide range of food products such as potato crisps, French fries or bread has been confirmed by Swedish scientists from Stockholm University. The neurotoxicity, possible carcinogenicity of this compound and its metabolites compels us to control them by quantitative and qualitative assays. Acrylamide forms adduct with hemoglobin (Hb) as a result of the reaction the -NH_2_ group of the *N*-terminal valine with acrylamide. In this work we present the use of glassy carbon electrodes coated with single-walled carbon nanotubes (SWCNTs) and Hb for voltammetric detection of acrylamide in water solutions. The electrodes presented a very low detection limit (1.0×10^-9^ M). The validation made in the matrix obtained by water extraction of potato crisps showed that the electrodes presented are suitable for the direct determination of acrylamide in food samples.

## 1. Introduction

Acrylamide is a well known neurotoxin and potential carcinogen [1-3]. High levels of this compound have been found in potato crisps, French fries and several other common foods [1, 4, 5]. The first such report was announced by scientists from Stockholm University in 2002 [1]. Acrylamide is formed in the reaction between reducing sugars such as glucose and the amino acid asparagine. The Maillard reaction mechanism has been proposed to account its formation in high starch foods during cooking at high temperatures [6-8]. Among analytical methods used for determination of acrylamide levels, expensive and time-consuming chromatographic techniques such as GC-MS [9], GC-MS-MS [10], HPLC-MS [11] and LC-MS-MS [5] predominate. Preparation of food samples for analysis involves extraction using water or methanol and the clean-up step typically consists of a combination of several solid-phase extractions. GC-MS often needs an additionally laborious bromination step to form a more volatile acrylamide derivative and increase the selectivity of the determination. Only a few examples using different techniques for detection of this toxic compound exist. Ignatov and co-workers prepared a biosensor in which respiratory activity of microbial cells was used for detection of acrylamide [12]. Kleefisch and co-workers reported a sensor in which acrylamide was detected at a gas–solid interface using an ‘electronic nose’-type quartz crystal microbalance (QCM) sensor covered with a tetralactam active layer [13].

It is known that acrylamide can create adducts with hemoglobin [1, 2, 14]. Hemoglobin (Hb) is the iron-containing oxygen-transport metalloprotein present in the red blood cells of vertebrates. Human Hb (MW=64,500) is an assembly of four globular protein subunits non-covalently bound to each other. Interactions between these subunits results in the allosteric properties of this protein. Each of the four subunits is composed of a protein chain tightly associated with a non-protein heme group, which is located in crevices near the surface of the molecule. These four iron-heme prosthetic groups are responsible for electroactivity of hemoglobin undergoing the reversible conversion of Fe(III) to Fe(II).

The Hb is susceptible to both reversible and irreversible modification: the sixth position of the iron can be spontaneously occupied by small ligands like oxygen, CO or CN^-^. The *N*-terminal amino acid of the β chain reacts spontaneously with glucose via an instable Schiffs base to ketoimine or fructose. The cysteine residues of the β chains are highly reactive and react with NO and other SH blocking reagents [15].

It is known that acrylamide and related conjugated vinyl compounds, undergo Michael-type nucleophilic addition reactions of amino (NH_2_) and sulfhydryl (SH) groups of amino acids, peptides and proteins to their double bonds [2]. Investigations showed that acrylamide-Hb adducts are formed as a result of the reaction between the α-NH_2_ group of the *N*-terminal valine of Hb with acrylamide [1, 2]. Therefore, hemoglobin can serve as useful biomarker of human exposure to acrylamide. The tracing of background exposure to acrylamide through biomarker measurements were conducted using GC-MS-MS method in the negative ion/chemical ionization mode [16] or with GC-MS [17]. Stobiecka and co-workers introduced a voltammetric sensor based on the reaction of hemoglobin with acrylamide [14]. This reaction led to the hemoglobin-acrylamide adducts formation, what alters the electroactivity of hemoglobin.

The adsorption of biomolecules directly onto the naked surfaces of bulk materials may frequently result in their denaturation and loss of bioactivity. The adsorption of such biomolecules onto the surface of nanoparticles can retain their bioactivity because of the biocompatibility of nanoparticles [18]. Carbon nanotubes (CNT) can serve as amplification platforms (‘carriers’) when loaded with numerous signal-generating molecules [19]. The SWCNT-modified electrode showed promising electrocatalytic behavior toward several biomolecules, such as dopamine, epinephrine and ascorbic acid [20]. Such modified electrodes might be used in biosensors to study the electrochemistry of biosystems. The subtle electronic properties suggest that CNT have the ability to promote electron transfer in electrochemical reactions, what has been documented in connection to importantbiomolecules such as hemoglobin, horseradish peroxidase and glucose oxidase [21].

In the literature we can find many examples of hemoglobin immobilization on the surfaces of gold, glassy carbon, pyrolytic graphite or carbon paste electrodes. Many procedures involve gold nanoparticles [22-28], single- and multi-wall carbon nanotubes [21, 29-32], whisker-like carbon composites [33].

Yin and co-workers reported biosensors in which hemoglobin, horseradish peroxidase and glucose oxidase were immobilized on the surface of carbon nanotubes modified glassy carbon electrodes (Nafion-Hb-CNT/GCE). The results obtained using cyclic voltammetry indicated that the redox protein and enzyme underwent effective and stable direct electron transfer reaction. The average value of the formal redox potential *E*^0^′ for Hb was –0.343 ± 0.001 V (vs. SCE, pH 6.9) [21].

R. Zhang and co-workers showed that Hb can be coupled to acid-treated multiwall carbon nanotubes (MWCNTs) in the presence of 1-ethyl-3-(3-dimethylaminopropyl) carbodiimide (EDC) and assembled as Hb-CNT composites on glassy carbon electrodes surfaces [29]. The electrochemical response of such prepared electrodes was compared with response of electrodes modified without using of EDC. These results indicate that use of EDC can accelerate the direct electron transfer of Hb effectively. Redox peaks were detected at -136 and -300 mV (vs. SCE, pH 5.0) and the peak current ratio at these potentials was about 1. When procedure without EDC was used, redox peaks detected were located in similar positions but electrochemical response of Hb was considerably lower.

Y. Zhang and co-workers investigated direct electrochemistry of hemoglobin immobilized on the DDAB/SWCNTs film modified gold electrode [31]. The immobilized Hb displayed a couple of redox peaks with the formal potential of about 129 mV (vs SCE, pH 7.0).

In this paper we present the use of glassy carbon electrodes coated with SWCNTs and Hb. In all experiments single-walled nanotubes (SWCNTs) were used as more stable and possessing less structural defects then MWNTs. The tubes performed a role of element promoting the electron transfer of Hb electrochemical reaction and ensuring its bioactivity. Hemoglobin was applied as an electroactive element able to react with acrylamide *in vivo* and on the surface of electrode. Such prepared electrodes were used for voltammetric detection of this analyte in water solution.

## 2. Materials and Methods

### 2.1. Materials

Hemoglobin human (Hb), acrylamide and potassium hexacyanoferrate(II) trihydrate were purchased from Sigma–Aldrich (Poznań, Poland). Acetic acid, zinc sulphate heptahydrate, sodium chloride, sodium hydroxide and hexane were obtained from POCh (Gliwice, Poland). Single-walled carbon nanotubes (SWCNTs) were purchased from CarboLex (Lexington, USA). Carrez I solution was prepared by dissolving potassium hexacyanoferrate (II) trihydrate (144 g) in deionized water (500 mL). Carrez II solution was prepared by dissolving zinc sulphate heptahydrate (288 g) in deionized water (500 mL). All aqueous solutions were prepared using freshly deionized water (18.2 MΩcm specific resistivity) obtained with a Simplicity 185 Water System (Millipore, Molsheim, France).

### 2.2. Preparation of electrodes

Glassy carbon electrodes (3.0 mm dia.) purchased from Bioanalytical Systems (BAS, West Lafayette, USA) were cleaned mechanically by polishing with wet 0.3 and 0.05 mm alumina slurry (Alpha and Gamma Micropolish; Buehler, Lake Bluff, USA) on microcloth pad (BAS) and by sonicating in water (30 sec.). Then the electrodes were dipped in 0.5 M H_2_SO_4_ solution and sweeping the potential between −300 mV and +1400 mV (versus a Ag/AgCl reference electrode) with scan rate of 100 mVs^−1^.

### 2.3. Purification of SWCNTs

Single-walled carbon nanotubes (SWCNTs, 10 mg) were purified by sonication for 30 min in a mixture of concentrated sulfuric and nitric acids (3:1 HNO_3_/H_2_SO_4_, 10 mL) and heating to reflux for 1 h. Afterwards, the resultant nanotubes were filtered and thoroughly washed using doubly distilled water and then dispersed in ultrapure water (1 mL).

### 2.4. Modification of electrodes

After the purification step the resulting solution of SWCNTs in water was diluted five times and 5 μL was dropped on the surface of glassy carbon electrode and left to dry at 4°C. Then 10 mg/mL hemoglobin solution in 0.2 M acetate buffer containing 0.05 M NaCl pH=5.0 (5 μL) was dropped on the electrode surface and again left to dry at 4°C. Then electrodes were stored on air at 4°C till use.

### 2.5. Electrochemical measurements

All electrochemical measurements were performed with an AutoLab potentiostat–galvanostat (Eco Chemie, Utrecht, Netherlands) with a three electrode configuration. Potentials were measured versus the Ag/AgCl reference electrode obtained from Bioanalytical Systems (BAS) West Lafayette, USA. Ag/AgCl wire was placed in a glass tube filled with 3 M NaCl. The tube was closed with a vycor plug to protect of the inner solution from direct contact with the sample. A platinum wire was used as the auxiliary electrode. Cyclic voltammetry (CV) was performed and potential was cycled from +400 to -400 mV with scan rate 100 mV/s for GCE coated with SWCNT and Hb (GCE/SWCNT/Hb). Osteryoung square wave voltammetry (OSWV) was performed in the same potential window with step potential of 1 mV, a square-wave frequency of 100 Hz, and amplitude of 25 mV. The dependence of the sensor response on the concentration of analytes was expressed as the currents at the peak potential in OSWV measured in a solution containing no analyte. The electrolyte compositions for the measurements was as follows: 0.05 M NaCl + 0.2 M acetate buffer, pH 5.0.

### 2.6. Preparation of potato crisp water extracts

Crushed potato crisps (40 g) were mixed in a mortar to obtain a homogeneous sample. The obtained powder was added to deionized water (400 mL) and the sample was stored for 20 min for swelling. After this incubation time, the sample was shaken for 1 h at 60 °C (Ika, HS-B20 digital) and then centrifuged for about 20 min at 4,500 rpm (MPW Med. instruments, MPW 350R). The supernatant solution was defatted by extraction with hexane. The obtained aqueous solution was purified by adding Carrez I and Carrez II (10 mL, see section 2.1). The supernatant was subsequently filtered. Such a prepared matrix was diluted 100-fold before electrochemical measurements. After that a suitable amount of concentrated acetic acid and NaCl were added to obtained concentrations 0.2 M and 0.05 M, respectively. pH of the final solution was adjusted to pH 5.0 with 1 M NaOH.

## 3. Results and Discussion

### 3.1. Characterisation of GCE/SWCNT/Hb electrodes

In this work we report an investigation concerning the electrochemistry of GCE electrodes modified with SWCNTs and Hb. The carbon nanotubes were used after consideration to their unique structural, mechanical and electronic properties [34]. The nanotubes were chemically treated with strong acids. This treatment not only purified them, but also reduced their length and led to the formation of carboxylic and phenolic groups at the nanotube's ends [35-37]. The acid treated SWCNTs were deposited onto surfaces of glassy carbon electrodes and than Hb was immobilized by dropping on SWCNT/GCE surface. The base of detection of acrylamide by proposed biosensor is the reaction of Hb-NH_2_ groups with the analyte. Because of this EDC was not used in the procedure of biosensor preparation. Involving Hb-NH_2_ groups in the reaction with SWCNT-COOH could decrease the yield of the Hb-acrylamide adduct formation.

**Figure 1.**
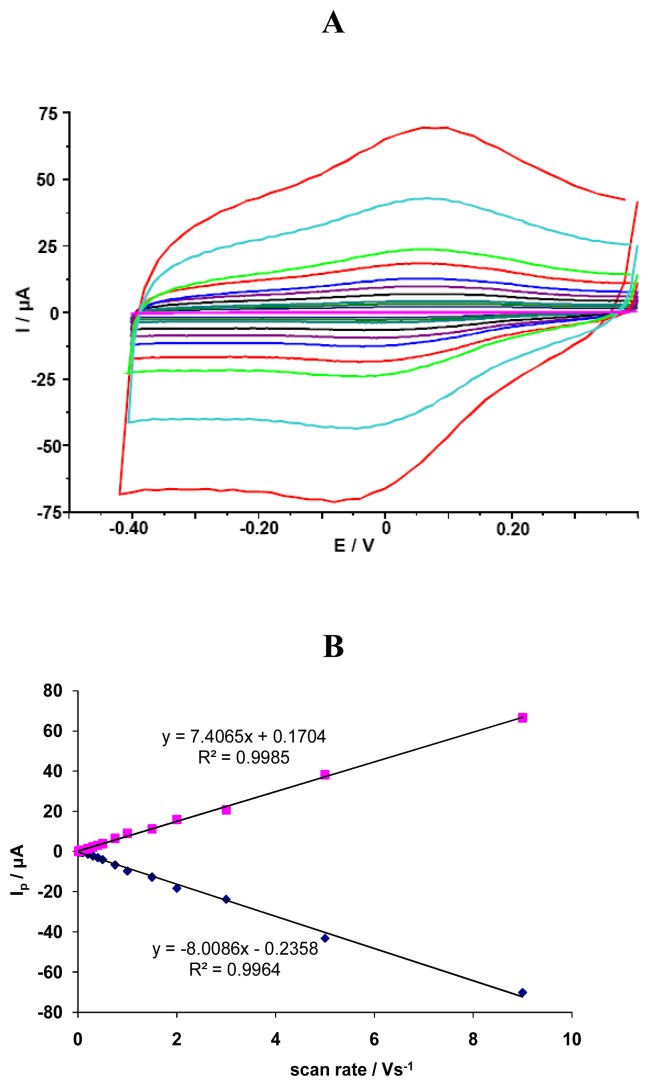
(A) The CV curves for GCE/SWCNT/Hb measured in solution containing NaCl 0.05 M and acetate buffer 0.2 M pH = 5.0 *vs* scan rates: 0.01, 0.02, 0.03, 0.04, 0.05, 0.075, 0.1, 0.2, 0.3, 0.4, 0.5, 0.75, 1.0, 2.0, 3.0, 5.0, 9.0 V/s. (B) Linear relationship between cathodic and anodic peak current *vs.* scan rate.

The investigated GCE/SWCNT/Hb electrodes displayed a quasi-reversible electrochemical reaction in measured solution pH=5.0 (Figure 1). The cathodic and anodic peak potential were located at *E*_cat_=72 mV and *E*_an_=141 mV, respectively. The difference between the cathodic and anodic peaks potential (ΔE_p_) was about 69 mV. The formal potential, estimated as the average of reduction and oxidation peak potentials, was about *E*^0^=107 mV. Cathodic *I*_p,c_ [μA] and anodic *I*_p,a_ [mA] peaks currents were linearly dependent on scan rate *ν*, ranging from 0.01 to 9.0 V/s (y=-8.008x-0.235, R²=0.996 for *I*_p,c_ and y=7.406x+0.170, R²=0.998 for *I*_p,a_) (Fig. 1). This indicates that redox reaction is not a diffusion-controlled process, but a surface-controlled one, as expected for an immobilized system [38].

Similar modification procedure was used by Qi and co-workers [30]. Acid-treated MWCNTs suspension in DMF was cast on the surface of glassy carbon electrode. After solvent evaporation Hb solution was dropped onto the surface of MWCNT/GCE. In the cyclic voltammograms of MWCNT/GCE a pair of redox peaks appeared at +67 mV and +110 mV (vs. Ag/AgCl, pH 5.4) and come from carboxylic groups of the functionalized MWNTs [20]. With Hb/MWCNT/GCE these peaks disappeared and a pair of new redox peaks appeared at -195 and -288 mV (measured in solution pH 5.4) which came from Hb. The formal potential was -240 mV. The disappearance of peaks at positive potentials after Hb immobilization was explain by interactions of Hb with carboxylic acid groups of the functionalized MWCNT.

Results reported in this paper were similar to those obtained by Qi and co-workers for GCEs modified only by MWCNTs [30]. Because of that electrodes modified only with nanotubes were investigated using OSWV. These electrodes (GCE/SWCNT) showed peaks in similar positions as compared with electrodes with Hb (GCE/SWCNT/Hb). After immobilization of Hb peak observed with OSWV become smaller, what is in good agreement with results reported by Qi and co-workers [30] and probably indicate interaction of Hb with carboxylic groups of nanotubes. In the case of our experiments, these interaction caused decreasing of peak current deriving from COOH, but not its disappearance.

After immobilization of Hb no peak appeared at negative potential when measured in solution saturated with argon. In solution containing small amount of oxygen (left in contact with air for some time after purging with argon) small peak appeared at about -300 mV connected probably with reduction of oxidized by oxygen hemoglobin. After purging the solution with inert gas, this peak disappeared. Such a phenomenon was not observed in the case of electrodes modified only with nanotubes (SWCNT/GCE).

We decided to check amperometric responses of electrodes modified with Hb (Hb/SWCNT/GCE) towards acrylamide and compare them with responses obtained for electrodes modified only with nanotubes (SWCNT/GCE).

### 3.2. Amperometric responses of SWCNT/Hb modified glassy carbon electrode towards acrylamide

**Figure 2.**
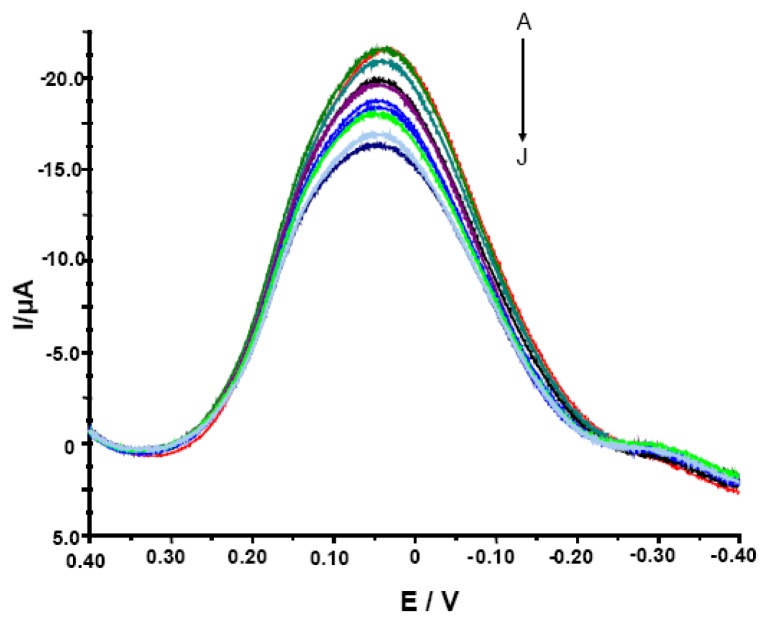
The OSWV curves for electrodes modified with SWCNT/Hb measured in the presence of various concentrations of acrylamide. The electrolyte composition: 0.05 M NaCl, 0.2 M acetate buffer pH = 5.0. The concentration of analyte was, as follows: (A) 0, (B) 1.0 × 10^−11^, (C) 1.0 ×10^−10^, (D) 1.0 × 10^−9^, (E) 1.0 × 10^−8^, (F) 1.0 × 10^−7^, (G) 1.0 × 10^-6^, (H) 1.0 × 10^-5^, (I) 1.0 × 10^-4^, (J) 1.0 × 10^-3^ M.

The determination of acrylamide by glassy carbon electrodes modified with single-walled carbon nanotubes and hemoglobin has been examined using two techniques: cyclic voltammetry (CV) and Osteryoung square wave voltammetry (OSWV) in solution containing 0.2 M acetate buffer pH 5.0 and 0.05 M NaCl. OSWV technique (Figure 2) proved to be much more sensitive in comparison with CV (data not shown).

It was expected since the OSWV has the ability to suppress of background currents. Therefore, this method was more sensitive and more suitable for quantitative measurements [39, 40]. Electrodes modified with SWCNT/Hb showed various peak current (from -4.8 to -21.6 μA) and peak potential (from 39.0 to 59.0 mV) in conditions of measurements and therefore, the parameter of relative decreasing of peak current was used to evaluate their responses. The ratio of OSWV peak current in the presence of different concentrations of acrylamide (I_p_) to that in the absence of analyte (I_p,0_) (I_p_/I_p,0_ x 100%) was plotted versus the acrylamide concentration. Even though the modified electrodes studied showed varied maximum current in measured solution, all of them displayed similar sensitivity towards acrylamide estimated with the use of the relative peak current decreasing (I_p_/I_p,0_ x 100%). Among numerous GCE/SWCNT/Hb studied, only 30% displayed weak or no response towards the analyte and had to be rejected. This let us to state that reproducibility of proposed biosensor is good.

The linearity of I_p_/I_p,0_ x 100% versus log c of acrylamide was observed in the concentration range from 1.0×10^-11^ to 1.0×10^−3^ M. The detection limit was estimated as 1.0×10^−9^ M taking into account a signal-to-noise ratio of 3 [41] and was low enough to detect acrylamide in food samples. Stobiecka and co-workers introduced a voltammetric sensor based on hemoglobin/DDAB liposomes dropped on the surface of carbon paste electrode [14]. These electrodes were used for detection of acrylamide in aqueous solution. Hemoglobin-acrylamide adducts were formed, what altered the electroactivity of hemoglobin and generated response of the biosensor. This biosensor displayed electrochemical response towards the analyte with similar linearity range of 1.3×10^−11^ to 5.6×10^−3^ M and sensitivity in comparison to biosensor proposed in this paper.

**Figure 3.**
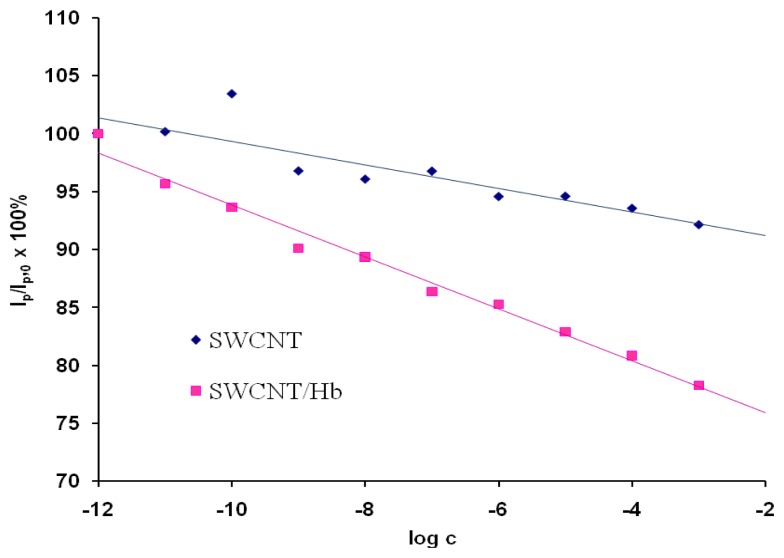
The ratio of OSWV peak current in the presence of different concentration of acrylamide (I_p_) to that in the absence of acrylamide (I_p,0_) as a function of the acrylamide concentration. The currents were measured at the peak potential in OSWV curves in the solution with no analyte E_p,0_. Results obtained for electrodes modified with SWCNT, E_p,0_=46 mV, n=4, 2.2<SD<7.7 ( 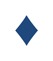) and SWCNT/Hb, E_p,0_=44 mV, n=3, 0.9<SD<4.4 ( 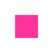).

As a control experiment electrodes modified only with acid treated SWCNTs were used These electrodes showed only negligible responses towards the analyte in the solution pH=5.0 (Figure 3). This proved that the response generation only resulted from the presence of Hb. The reaction of hemoglobin-acrylamide adducts formation alters the electroactivity of investigated system.

The electrodes modified with Hb were very stable. After preparation, they could be stored on air (at 4 °C) ca. 2 weeks (a longer period was not explored). The interaction between Hb and acrylamide was irreversible. Therefore, after contact with acrylamide solution, the electrodes had to be cleaned and modified again. Because of this, the repeatability of investigated electrodes could not be estimated. However, the modification procedure was very simple, what is one of the most important advantages of presented biosensor.

### 3.3. Detection of acrylamide in the presence of water extract from potato crisps using a glassy carbon electrode modified with hemoglobin

**Figure 4.**
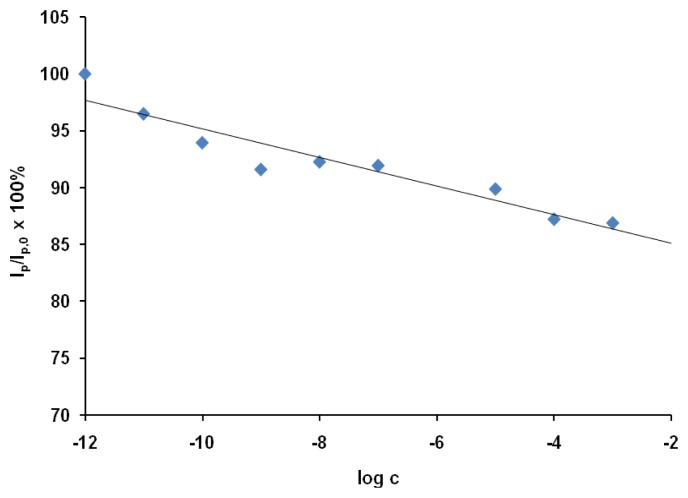
The response of Hb/SWCNT/GCE electrodes towards acrylamide in the presence of water extract from the potato crisp. The ratio of OSWV peak current in the presence of a given concentration of acrylamide (*I*_p_) to that in absence of analytes (*I*_p,0_) was plotted versus the concentration of acrylamide in water extract from the potato crisp. The currents were measured at the peak potential in OSWV curves in the solution with no analyte (*E*_p,0_ = 38 mV); (*n* = 3; 3.1 < S.D. < 7.5).

The applicability of the proposed sensor was tested using the OSWV technique, which was more sensitive than CV. The responses of the GCEs modified with SWCNT/Hb towards acrylamide were measured in the presence of sample solutions based on 100-fold diluted water extracts from potato crisps. The sample solution also contained 0.2 M acetate buffer (pH 5.0) and 0.05 M of NaCl. The relation of relative decreasing of peak current versus log c was shown in Figure 4.

The electrodes modified only with SWCNT (without Hb) responded towards the analyte weakly. 1 mM acrylamide in the synthetic solution generated only 7.9 ± 6.8% current decrease. Therefore this type of electrodes was not checked in the presence of the matrix.

The presence of the matrix obtained from potato influenced slightly on the electrode sensitivity towards acrylamide. The limit of detection estimated in the presence of the potato crisp matrix defined from a signal-to-noise ratio of 3 [41] was 1.0×10^−9^ M. The linear range of this response was from 1.0×10^−11^ to 1.0×10^−3^ M.

There is no significant difference between analytical parameters of response towards acrylamide such as linear range or detection limit obtained for Hb/SWCNT/GCE in aqueous solutions of acrylamide and in potato crisp extract, but the response measured in the synthetic solution was stronger. The highest acrylamide concentration studied (1.0 x 10^-3^ M) caused 21.7 ± 3.5% and 13.0 ± 3.0% current decrease measured using OSWV, in the synthetic sample solution and in the potato crisps extract, respectively.

A comparison of results obtained in the presence of the potato crisp matrix (Figure 4) with those obtained in synthetic solution (without the matrix - Figure 3), allows us to state that the sensor under study is resistant to interference coming from the matrix obtained by extraction of potato crisps. The next advantage is very good sensitivity in the 10^−9^ M range. Also, the application of the proposed sensor for acrylamide determination does not require complex sample preparations.

The weak influence of the natural matrix on the electrodes sensitivity towards acrylamide proved their resistivity to interference coming from the potato crisps water extract. Thus it might be concluded that proposed biosensor can be used for determination of the level of acrylamide in such prepared extract.

## 4. Conclusions

Glassy carbon electrodes coated with SWCNT and Hb showed good voltammetric response towards acrylamide. The chemical base of the proposed biosensor is the formation of Hb-acrylamide adduct by the Michael-type nucleophilic addition reaction of a valine -NH_2_ group to the acrylamide double bond.

The observed redox reaction is not a diffusion, but rather a surface-controlled process. Observed redox peaks probably result from the COOH groups of SWCNTs, not from Hb, but reaction of Hb-NH_2_ with acrylamide alters the redox behaviour of the investigated system.

Osteryoung square wave voltammetry (OSWV) was more suitable for quantitative determination of acrylamide with electrodes presented than cyclic voltammetry (CV). The presence of natural matrix obtained by water extraction from potato crisps influence only little on the acrylamide determination with glassy carbon electrode modified with hemoglobin. The biosensor display low detection limit (10^-9^M) and wide dynamic range. Its preparation is relatively simple and inexpensive. Therefore, the proposed biosensor might be recommended for the direct determination of the acrylamide in the food samples.

## List of abbreviations

CNT: carbon nanotubes
CV: cyclic voltammetry
DDAB: dimethyldioctadecyl-ammonium bromide
DMF: dimethylformamide
EDC: 1-ethyl-3-(3-dimethylaminopropyl) carbodiimide
GCE: glassy carbon electrode
GCE/SWCNT/Hb: glassy carbon electrodes modified with single-walled carbon nanotubes and hemoglobin
GC-MS: gas chromatography-mass spectrometry
GC-MS-MS: gas chromatography-tandem mass spectrometry
Hb: hemoglobin
HPLC-MS: high-performance liquid chromatography-mass spectrometry
LC-MS-MS: liquid chromatography- tandem mass spectrometry
MWCNTs: multiwall carbon nanotubes
Nafion-Hb-CNT/GCE: glassy carbon electrode modified with carbon nanotubes, hemoglobin and Nafion
OSWV: Osteryoung square wave voltammetry
QCM: quartz crystal microbalance
SCE: saturated calomel electrode
SWCNT-COOH: acid treated single-walled carbon nanotubes
SWCNTs: single-walled carbon nanotubes
